# Hydrogen Adsorption on Nearly Zigzag-Edged Nanoribbons: A Density Functional Theory Study

**DOI:** 10.1038/s41598-017-14189-z

**Published:** 2017-11-16

**Authors:** Michael Rivera Mananghaya, Gil Nonato Santos, Dennis Yu, Catherine Stampfl

**Affiliations:** 1 Ateneo de Manila University, Katipunan Ave, Quezon City, 1108 Metro Manila Philippines; 2 De La Salle University, 2401 Taft Avenue, 0922 Manila, Philippines; 3 NRCP (IX), DOST, Gen. Santos Ave., Bicutan Taguig City, 1631 Philippines; 4 University of Sydney, Sydney, NSW 2006 Australia

## Abstract

The realistic shapes of N doped graphene nanoribbons (GNRs) can be realized by considering nearly zigzag-edged (NZE) imperfections and pyridine defects (3NV). The paper focuses on NZE-GNRs with 3NV that is populated by Scandium abbreviated as Sc/NZE-3NVGNRs. Systematic calculations have clarified roles of the nano-shapes of NZE-3NVGNRs in its formation, energetics, stability and electron states functionalized with Sc using density functional theory (DFT) formalisms. According to DFT calculations, the magnitude of the spin that is attributed to the rise of magnetic order is closely linked to the altered shape of the ribbon edges. Also, calculations show that the stability of Sc functionalization at the 3NV and NZE site is thermodynamically stable and is dictated by a strong binding energy (BE). The magnitude of the BE is enhanced when the zigzag edge is short or the ribbon width is narrow, suggesting a reduced clustering of Sc atoms over the Sc-doped NZE-3NVGNRs. Results also show that as the length of the zigzag edge in Sc/NZE-3NVGNRs increases it creates considerable distortion on the appearance of the structure. Finally, the Sc/NZE-3NVGNRs as a potential candidate for hydrogen storage was evaluated and it was found that it could adsorb multiple hydrogen molecules.

## Introduction

Graphene has attracted numerous scientific investigations due to its remarkable physical, mechanical, electronic and magnetic properties^1–8^. The graphene’s inherent robustness and lightness render the novel carbon nanomaterials to be prominent materials in the frontiers of science and engineering applications. In particular, electrons near the Fermi level in graphene adhere to the massless Dirac equation, leading to linear dispersion in the low-energy regime of the excitation spectra^8,9^. Two dimensional (2D) graphene sheets, when cut into rectangle slices, can become one-dimensional (1-D) semiconductors with unusual electron states localized at its edges called graphene nanoribbons (GNRs)^10–12^. The GNRs energy band gap is deeply rooted on the width and crystallographic orientation as predicted by tight-binding (TB) calculations. The TB shows a flat band situated at the fermi level for zigzag GNR (ZGNR) whose wave function is localized near the edge. Further calculations have verified this discovery that demonstrates also the absence of a flat band for armchair GNR^12^. Later, the local density of states (DOS) of the zigzag edges measured by microscopy confirms the existence of these local edge states^13,14^. The novel perspectives and research on GNR is currently developed for semiconductor devices that exhibits ferromagnetism in its zigzag-edge^15–17^. Actually, a density-functional theory (DFT) calculation with correlation effects treated by the generalized gradient approximation (GGA) scheme–has confirmed that the spin is polarized ferromagnetically on the zigzag edge of GNR such that it is coupled in an anti-ferromagnetic way^15–17^. Based on these theoretical results, the rise of novel GNR in spintronic applications with half-metal properties has been reported^17^.

An ideal substitute for fossil fuel-coal, natural gas and oil is H_2_ as it is non-toxic and environmentally friendly. Pressurized tank, liquid or solid phase materials can be used as storage media although, a very few satisfy the requirements of safe and cost-effective storage media required for H_2_ powered systems. The storage capacity or operating conditions fall below the standard set by the Department of Energy (DOE) of >6 wt.%. It is recommended by the U.S. DOE that for room temperature application of hydrogen energy, the optimal adsorption enthalpy should be within ~0.1–0.2 eV per H_2_ at ~30 bar^15–17^. Furthermore, carbon-based related nanostructures, such as fullerenes, carbon nanotubes, graphene, GNRs have been explored as adsorbent media for hydrogen gas because of their large surface area per unit weight. Interestingly, Nitrogen (N) clusters found in nitrogen doped zigzag-edged GNR^15–22^ can significantly modify the band structure of graphene and nanoribbons and can facilitate an improved hydrogen adsorption^17^. Specifically, pyridine vacancies (3NV) are also found in the zigzag edges because of the unzipping process of N doped carbon nanotubes (CNTs). These unzipping offers another approach of producing zigzag-edged GNR with N doped vacancies. However, straight unzipping is not physically realizable at present and nearly zigzag-edged GNRs (NZE-GNRs) with 3NV vacancies (NZE-3NVGNR) exist^22–26^. Thus, it is imperative to decipher the NZE-3NVGNR robustness and its relative stability theoretically.

On the other hand, an adsorbed metal can act as active adsorption site for H_2_. However, several Alkali or alkali earth metal (Li, Na, K, Ca) decorated nanomaterials may lead to relatively low adsorption energy. Moreover, transition metal (TM) decorated nanostructured materials with Ti and V can yield higher adsorption energy^17–20^ due to interactions which result from the hybridization of the sigma and sigma* orbitals of H_2_ with transition metal *d* orbitals. The TM decorated systems tend to form clusters rather than spreading over the adsorbent, resulting in metal aggregation and consequently reduced hydrogen storage capacity. The strong TM cohesion is responsible for such aggregation. To overcome the formation of metal clusters, it is strongly suggested to increase the binding strength between TM and GNR by introducing 3NV and edge shape imperfections^10,11^. The TM Scandium once incorporated holds promise for the design of lightweight reversible adsorption systems for H_2_ storage at room temperature. For the number of H_2_ to be adsorbed on each Sc-decorated ideal zigzag-edged (IZE) nanoribbons with 3NV defects, rigorous calculation shows that it is around five^17^. Furthermore, in the IZE-GNR, the valence-band maximum (VBM) and the conduction-band minimum (CBM) states are degenerate, characterized by a linear combination of *ϕ*_*l*_ and *ϕ*_*r*_ pi orbitals on the left and the right edges, respectively^17^. Here, a systematic study of electronic structures of NZE-3NVGNR decorated with Scandium is presented. The Sc functionalized NZE-3NVGNR are demonstrated below to act as a suitable candidate for hydrogen storage. The purpose of the present paper is to clarify the intricate roles of Nano shapes of the edges in Sc functionalized NZE-3NVGNR in its energetics and H_2_ adsorption. GNRs having finite-length zigzag edges was subjected to total energy electronic-structure calculations. The formation energy, stability of Sc binding to the NZE-3NVGNR and electron states with various edge shapes are also discussed clearly and completely.

## Computational

**Figure 1:**
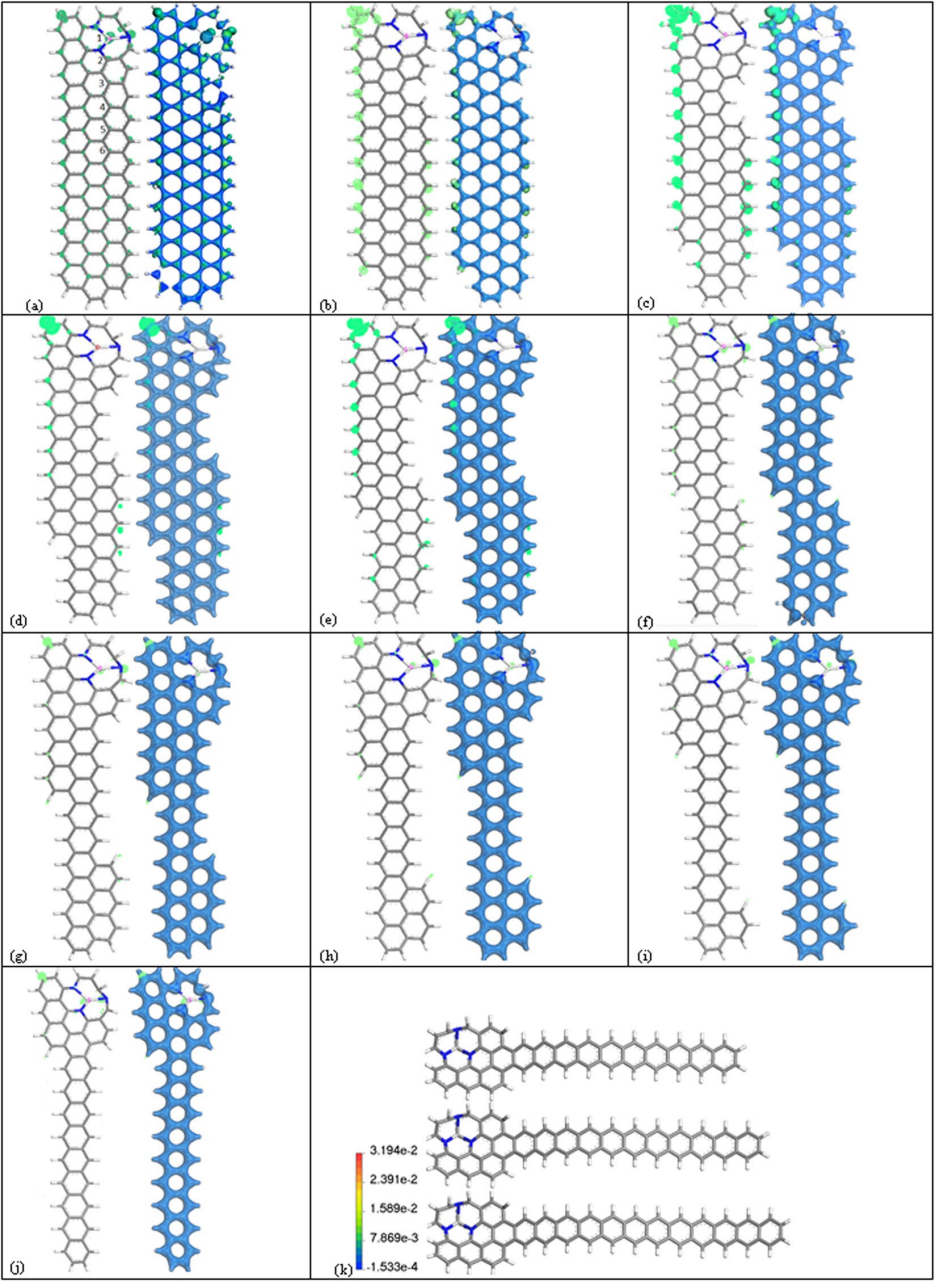
Structure and Spin of (**a**) NZE-3NVGNR-Spin distribution localized in the edges (green regions); NZE-3NVGNRs with n_zig_ starting from (**b**) one to (**j**) nine with the Sc-N_3_ center highlighted in pink. The blue contour is the total electron density; the Spin in the left structure is embedded in the succeeding density figures on the right side and eventually fades as n_zig_ progresses. The model was generated in such a way that as n_zig_ increases, the width decreases. In addition, an elongated tail is exhibited for (k) n_zig_ = 10, 11, 12 with N-blue, C-gray and H-white.

To unravel the relation between the Nano shapes and the electronic/magnetic properties in graphene systems, first-principles DFT calculations are carried out via the GAMESS code^27^. The structural optimizations and natural bond orbitals (NBO) analysis were performed using the improved generalized gradient-corrected Perdew-Burke-Ernzerhof (PBE/GGA) functional^28^ employing an empirical dispersion part with 6–31 G (d, p) basis set for the spin-unrestricted DFT computation. The implemented empirical dispersion correction further improves the long-range behavior of DFT methods. However, since GGA calculations tends to underestimate the HOMO-LUMO gaps of GNRs, the hybrid B3LYP-D was utilized in the gap calculation^29^. Two types of modified ZGNRs were considered: substitution of nitrogen dopants, by removing a single C atom among three hexagons and replacing the three surrounding C atoms with 3 N atoms (3NVGNR) (see Fig. 1a for the C_100_ScN_3_H_32_ cluster model) and NZE-3NVGNRs with length of the zigzag edge (n_zig_) varying from Fig. 1b one to Fig. 1j nine with the center of the 3NV defect functionalized by a Sc atom. In Fig. 1a to j the 3NV defect was populated by a Sc atom, for a detailed study on 3NVGNR with perfectly straight edges with and without Sc doping please consult Ref.^17^. The basis set superposition error (BSSE) correction approach was incorporated in the density functional dispersion correction (DFT-D) scheme, which further improves the accuracy in evaluating weak interactions. The DFT semi-core pseudo-potentials (DSPPs) were employed to efficiently treat with the core electron of the Scandium metal. The structures were viewed using the discovery studio visualizer^30^ a visualization tool for viewing and analyzing modeling data. The system was optimized until the force on each atom during relaxation was less than 0.03 eV/Å.

To properly address edge energetics, the edge formation energies (E_edge_) was calculated as given by the equation:

1 $${{\rm{E}}}_{{\rm{edge}}}=({{\rm{E}}}_{{\rm{rib}}}\,-\,{{\rm{N}}}_{{\rm{C}}}{{\rm{E}}}_{{\rm{C}}}\,-\,{{\rm{N}}}_{{\rm{H}}}{\mu }_{{\rm{H}}}\,-\,{{\rm{N}}}_{{\rm{N}}}{\mu }_{{\rm{N}}})/{{\rm{N}}}_{{\rm{C}}}$$

where E_rib_, N_C_, E_C_, N_H_, *μ*_H_, N_N_, *μ*_N_ represent the total energy for the nanoribbon ground states, the number of C atoms, the total energy of graphene per atom, the number of H atoms, the chemical potential of H atoms, the number of N atoms, the chemical potential of N obtained from the gas phase, respectively all obtained through GGA calculation. Thermodynamic stability of an edge in the presence of gas molecules should take the energetic cost of forming the edge into account. The Chemical potential calculated is at 300 K and atmospheric partial pressure for the hydrogen and nitrogen gas considered that is valid within accessible experiments. The chemical potential of the N_2_ gas obtained from ${{\rm{\mu }}}_{{{\rm{N}}}_{2}}$ = ½(${{\rm{E}}}_{{{\rm{N}}}_{2}}$ ^DFT^ + ${{\rm{E}}}_{{{\rm{N}}}_{2}}$ ^ZPE^ + ${{\rm{\mu }}}_{{{\rm{N}}}_{2}}$ ^0^ + RT*ln* (${{\rm{P}}}_{{{\rm{N}}}_{2}}$))^31^ where ${{\rm{E}}}_{{{\rm{N}}}_{2}}$ ^DFT^ is the DFT energy, ${{\rm{E}}}_{{{\rm{N}}}_{2}}$ ^ZPE^ is the zero-point energy, ${{\rm{\mu }}}_{{{\rm{N}}}_{2}}$ ^0^ is the chemical potential at the standard pressure which can be obtained from standard thermodynamic tables and ${{\rm{P}}}_{{{\rm{N}}}_{2}}$ is the equilibrium partial pressure of the N_2_ gas. It is understandable that the atmospheric partial pressure of ${{\rm{P}}}_{{{\rm{N}}}_{2}}$ is 0.781 atm. The resulting chemical potential for hydrogen is constructed similarly as ${{\rm{\mu }}}_{{{\rm{H}}}_{2}}$ = ½(${{\rm{E}}}_{{{\rm{H}}}_{2}}$ ^DFT^ + ${{\rm{E}}}_{{{\rm{H}}}_{2}}$ ^ZPE^ + ${{\rm{\mu }}}_{{{\rm{H}}}_{2}}$ ^0^ + RT*ln* (${{\rm{P}}}_{{{\rm{H}}}_{2}}$)). However, since there is almost no hydrogen gas in air, the ${{\rm{P}}}_{{{\rm{H}}}_{2}}$ is taken as ½ part per million, this corresponds to a ½(${{\rm{\mu }}}_{{{\rm{H}}}_{2}}$ ^0^ + RT*ln* (${{\rm{P}}}_{{{\rm{H}}}_{2}}$)) equivalent to −0.386 eV which accompanies the reaction in the sorbent material. The spin polarization S is defined as ${\rm{S}}=\int dr| {n}_{\uparrow }(r)\,-\,{n}_{\downarrow }(r)|$ where ${n}_{\uparrow }(r)$ and ${n}_{\downarrow }(r)$ represent the up and the down spin densities, respectively. The S is calculated in order to examine the existence and magnitude of the spin polarization for the various edge shapes of the NZE-3NVGNR in a concise and systematic way. The calculated ground states of ZGNRs are singlets for all the widths investigated. The optimization scheme carried out for several spin multiplicities are compatible with the number of electrons. The total quantum spin of zero corresponding to the singlet is the most stable fundamental state. Due to a negligible singlet-triplet energy, the transition from singlet to triplet state is instantaneous. Accordingly, the optimization scheme suggests that an actual graphene nanoribbon has a ground state with nearly degenerate singlet and triplet states. Since the singlet has a net magnetic moment of zero the spin multiplicity of the NZE-3NVGNR is set at triplet state for further calculations. The binding energy (BE) per Scandium atoms along the edge is given as

2 $${{\rm{E}}}_{{\rm{b}}-{\rm{Sc}}}=({{\rm{E}}}_{{\rm{rib}}+{\rm{Sc}}}\,-\,{{\rm{E}}}_{{\rm{rib}}}\,-\,{{\rm{N}}}_{{\rm{Sc}}}{\mu }_{{\rm{Sc}}})/{{\rm{N}}}_{{\rm{Sc}}}$$

where E_b-Sc_ is the BE of Sc atoms with the NZE-3NVGNR, E_rib+Sc_ is the total energy for the nanoribbon functionalized with Sc atoms, E_rib_ is the total energy for the nanoribbon, N_Sc_ is the number of Sc atoms, *μ*_Sc_ is the chemical potential for Sc atom derived from a large Sc_96_ cluster all in ground states using spin-unrestricted GGA calculation. The cluster was constructed using a hexagonal close-packed crystal structure as its basis sufficiently large enough to ignore the edge and boundary effects.

## Results and Discussions

### Electronic properties of Sc decorated NZE-3NVGNR

The calculated E_edge_ for various configurations of the NZE-3NVGNRs shown in Fig. 1 is tabulated in Table 1 as a function of n_zig_. The formation energies increase according to Table 1 when the NZE-3NVGNRs have longer zigzag edges. The model was constructed such that the NZE-3NVGNR with n_zig_ = 1 correspond to an armchair-3NVGNRs and n_zig_ = ∞ corresponds to zigzag-3NVGNRs.

**Table 1:** The edge formation energy (E_edge_) and spin (S) for successive increase of n_zig_, binding energy per Sc atoms within the NZE-3NVGNR (E_b-Sc_), HOMO-LUMO gap (E_gap_), charge transferred from Sc to the nanoribbon (C_Sc_), adsorption energy of H_2_ within the Sc functionalized NZE-3NVGNR (E_ads_), charge transferred from Sc to the nanoribbon in the presence of a H_2_ (C_Sc-H2_) and the average H-H distance (D_H2_). The DFT-D scheme was utilized to describe the van der Waals (vdW) interaction with BSSE correction. All entries were calculated using the GGA except the E_gap_ wherein B3LYP functional was employed.

n_zig_	E_edge_ (meV/edge)	S (Å^−1^)	E_b-Sc_ (eV)	*E_b-Sc_ (eV)	E_gap_ (eV)	C_Sc_ (e)	E_ads_ (eV)	*E_ads_ (eV)	C_Sc_-_H2_ (e)	D_H2_ (Å)
1	101.019	0.188	−3.438	−3.153	0.082	0.316	−0.249	−0.189	0.320	0.770
2	159.362	0.185	−3.978	−3.662	0.146	0.318	−0.256	−0.198	0.323	0.771
3	202.295	0.177	−4.025	−3.681	0.164	0.316	−0.257	−0.194	0.323	0.771
4	210.660	0.172	−4.541	−4.194	0.201	0.309	−0.307	−0.238	0.319	0.771
5	207.743	0.163	−4.810	−4.402	0.209	0.307	−0.251	−0.208	0.318	0.772
6	207.761	0.157	−5.152	−4.706	0.246	0.303	−0.283	−0.219	0.312	0.771
7	207.848	0.151	−5.200	−4.771	0.274	0.289	−0.254	−0.199	0.304	0.773
8	207.915	0.145	−5.270	−4.802	0.291	0.276	−0.275	−0.197	0.291	0.773
9	202.444	0.140	−5.392	−4.923	0.348	0.262	−0.283	−0.218	0.281	0.778
10	211.983	0.133	−5.413	−4.997	0.336	0.245	−0.312	−0.239	0.266	0.783
11	241.165	0.127	−5.386	−5.016	0.382	0.226	−0.356	−0.279	0.249	0.789
12	287.934	0.121	−5.309	−5.035	0.411	0.205	−0.415	−0.337	0.230	0.798

In S the Planck constant is usually dropped and has a unitless value divided by the corresponding length of the n_zig_ in Å.

*E_b-Sc_ is the binding energy of Sc atoms to GNRs and *E_ads_ is H_2_ adsorption to Sc-decorated GNRs both with H-termination. E_ads_ is obtained by averaging several model configurations with a standard deviation of $\pm$0.003 eV.

The present results verify the previous study in which it was proven experimentally that armchair-edged GNRs are more stable than zigzag-edged GNRs under normal synthesis conditions^23^. It reveals that as n_zig_ is changed from 1 to 12, the E_edge_ increases from 101.02 meV to 287.93 meV/edge atom, numerically close to the value of zigzag-edged at 285 meV/edge atoms. Here it can be reiterated that the difference between the armchair-edged GNR and the zigzag-edged GNR is 186 meV/edge atoms and this value is close to ~200 meV/edge atom as reported before^23^. The discrepancy in Eedge is due to the 3NV defect inherently added in the working model to depict reality in experimental conditions. The DFT calculations presented here indeed show that for N doped GNRs, the zigzag-GNR is less stable in energy by around 0.2 eV per edge atom than the armchair-GNR. Therefore, the N doped zigzag edges are less frequently encountered. However, it is worth mentioning that non-equilibrium conditions with relatively high temperature syntheses conditions and possible use of catalysts can produce zigzag edges with 3NV vacancies of finite length. In addition, as stipulated in Table S1 the E_edge_ decreases as the width (*w*) of the ribbon increases. Here *w* denotes the label for a GNR with *w* zigzag chains as *w*-ZGNR. This further indicates that narrower ribbons are relatively less stable in energy compared to wider zigzag nanoribbons. The smaller value specifies that the particular structure is more physically realizable. However, for *w* > 5, the edge formation energy approaches a limiting value near 80–90 meV/edge. In particular, the 10-ZGNR system resembles an infinite graphene sheet and consequently the effect of the edges is negligible, it corresponds to the most stable structure. Hence, structures with lower *w* are particularly reactive ZGNRs.

The location of the Sc-N_3_ center highlighted in pink in Fig. 1 produces an affect in the formation energy. According to S2 the impurities near the edge of the GNR plays an important role in the edge formation energy of the system. The E_edge_ of GNRs is sensitive to the location of the 3NV center where Sc adsorption takes place. The E_edge_ increases as the Sc-N_3_ center moves from position 1 to 6. Early calculations^32^ demonstrated that the spin-up and spin-down bands of narrow width isolated ZGNR are fully degenerate; the anti-ferromagnetic (AFM) phase has a zero net magnetic moment that is accompanied by a finite energy gap. Upon the introduction of a 3NV defect, half-metallic properties emerge depending on the configuration of N–C bonds of the pyridine defect and the outermost C atom of the ZGNRs. The bands near the Fermi level is asymmetric: in the spin-up channel, a metallic nature emerge, while the spin-down channel is semiconducting. Further, the 3NVGNR is still asymmetric with an N-type semiconducting feature after the adsorption of Sc atom strategically located within the center of the 3NV. Similarly, the spin-up and the spin-down states of a narrower nanoribbon shown in Fig. 1a possess asymmetric ordering. The Sc-N_3_, which are unevenly distributed in the ZGNRs edges can produce a net magnetic moment.

In the NZE-3NVGNRs model, the VBM and CBM states can be modelled with linear combinations of *ϕ*_*l*_ and *ϕ*_*r*_ as discussed briefly in the introductory section. The Kohn–Sham (KS) orbitals for the representative models of NZE-3NVGNRs are generally allocated near the edges. The penetration of the edge states into the middle area of the ribbon amplifies the extent of hybridization between the two edge states. The nano-shape of the ribbon of NZE-3NVGNR along with the separation of the left and right edges are principal factors to determine its electronic structure. The representative NZE-3NVGNRs from n_zig_ = 1 to 9 in which their ground states are all spin-polarized are shown also in Fig. 1b to j. The calculated spin-up and the spin-down states of the KS orbitals shows an anti-ferromagnetic ordering in NZE-3NVGNRs with respect to various zigzag length. The spin-polarization obtained here is consistent with the observed magnetism theoretically^15,16^. The included value of S in Table 1 for various NZE-3NVGNR as a function of the length of the zigzag edge n_zig_ is now discussed. As shown visually from the model structures, the NZE-3NVGNR with shorter zigzag edges possess a wider width which causes spin polarization due to the hindering of the hybridization of the two waves *ϕ*_*l*_ and *ϕ*_*r*_ from the two sides far from each other, furthermore a larger n_zig_ corresponding to narrow ribbon width acts conversely; it decreases the intensity of spin polarization. The result correlates well with a Local Spin Density Approximation (LSDA) calculation for chiral NZE-GNRs to be published soon. Additionally, the n_zig_ is an important factor to consider for the fine-tuning of anti-ferromagnetic spin polarization that depends on the width of NZE-GNR. A more detailed and rigorous calculation was performed to verify this claim by increasing the width of the ribbon and results indeed follow the argument. Thus, it can be deduced now that magnetism confined along the edge of zigzag-edged GNRs is a robust property based on quantum mechanical effects, as exemplified by the models presented. However, the random orientation of the edges in reality does not allow full access to the nature of edge magnetism. Though magnetic order is allowed to persist to some extent on zigzag segments of randomly oriented edges in the GNR structure, the mixing of different edge types and possible strong edge-substrate hybridization are expected to substantially weaken the effect. It is clear however that the modification of crystallographic orientation of graphene edges allows an overall control over both electronic and magnetic properties of GNRs.

**Figure 2:**
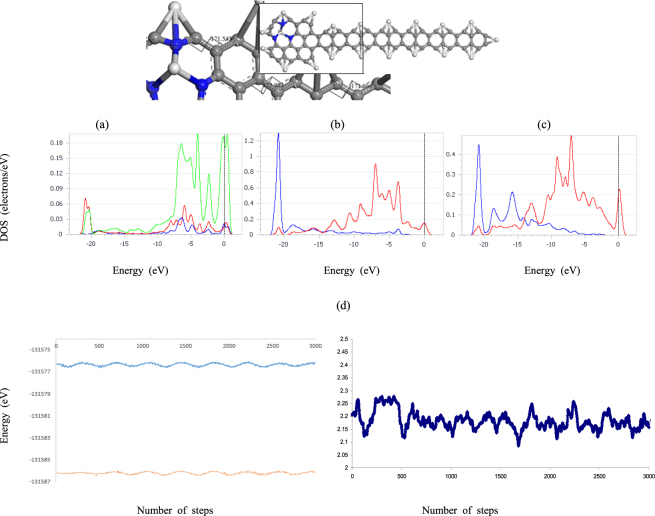
The relaxed structure of the Sc/NZE-3NVGNR system for n_zig_ = 12. The zigzag edge was distorted from its ideal 180° angle measure. The Partial Density of States of the ScN_3_ center with (**a**) Sc and (**b**) N orbitals. The (**c**) orbital of the Carbon attached directly to Sc of the NZE-3NVGNR. The blue, red and green curves denote s, p and d orbitals, respectively. The unit of the vertical axis is in electrons/eV and the horizontal axis is in eV. Molecular dynamics simulation of the total energy in eV at (**d**) 300 K (orange) and 500 K (blue) along with the bond length in Å of the Sc-C bond.

The relaxed structure of the GNR have edge atoms that possess electrons with highly delocalized acceptor like states^17^ along the edge that can lead to a stronger binding of TM such as Scandium atoms, this is also true for the NZE-3NVGNR calculated using equation (2). The absolute value of the BE per Scandium atom along the edge of the model increases as n_zig_ increases. Thus, the relative stability of the Sc-C bond increases as the atomic scale structural defects progresses. More importantly, these suggests that Sc bonded with carbon atoms at the NZE-3NVGNR edge is thermodynamically stable. In other words, edge-decorated NZE-3NVGNR by Sc is energetically robust. Another question to be addressed is do Sc atoms prefer to bind with carbon atoms on the surface of GNR? The Sc-C bonding scheme as depicted in Fig. 2 indicates that if Sc atoms are uniformly dispersed on the surface of graphene it migrates until it reaches the C atoms near the edge as observed in a dynamics simulation discussed below. Also, calculations indicate that if each Sc atom located at the center of a carbon hexagon is fixed, the Sc binding energy is merely 2.1 eV per Sc atom; it is inherently lower compared to the cohesive energy (E_coh_) of the bulk Sc metal. A BE less than E_coh_ suggests that clustering on the surface of GNR is prominent. The edge C atoms offer a strong influence due to edge orientation and can disperse Sc uniformly for n_zig_ = 4 (n_zig_ = 5 with GNRs with H-atom termination) since the magnitude of E_b-Sc_ equivalent to 4.541 eV is greater than the E_coh_ = 4.4 eV of Sc. If a moderate level of edge decoration is realized on the edge as n_zig_ increases, the adsorbed Sc has an average bond length of around 2.199 Å as depicted in Fig. 2 wherein the edge of the Sc/NZE-3NVGNR structure in Fig. 1 for n_zig_ = 12 is saturated with the lightest transition metal.

The Partial Density of States of the ScN_3_ center in Fig. 2a,b denotes that the binding states situated on the Fermi level is attributed to the Scandium *d* orbital hybridized with the *p* orbital of Nitrogen atom. This is reasonable, because the 3NV uses two valence electrons to form a lone pair. The hybridization of the Carbon *p* orbital in Fig. 2c and the Scandium *d* (shown in Supplementary Fig. S1, similar to Fig. 2a with a more pronounced Sc atom 3*d* state) orbital contributes to the strong interaction between Sc and the 2p states of the neighboring carbon atoms of the NZE-GNR. This results in the bonding of the Sc atom with the edge of the GNR, which is also true for the Scandium *d* and Nitrogen *p* orbitals of the Sc-3NV located near the edge as mentioned earlier. The large Sc binding energy at the edges is due to the charge transferred from the Sc to the NZE of the nanoribbon. These findings are similar to ref.^17^ as exemplified by ZGNR with ten zigzag chains (10-ZGNR). In addition, NZE-3NVGNR may adsorb fewer Sc atoms than those corresponding to structures with curvatures, accordingly binding energies of Sc atom with other low-dimensional carbon nanostructure, e.g. C_60_ and (10,0) single-walled carbon nanotube (SWCNT) were computed in consistent comparison with previous results^32–34^. The calculated binding energies between Sc and C_60_, Sc and 10-SWCNT are less than −2.5 eV, which is considerably less than the E_coh_ of bulk Sc as with the GNR surface case. Also, the examined binding energies of Sc atom with a Stone-Wales (SW) and a vacancy defect on (10,0) SWCNT were elucidated. A SW defect gives rise to a BE of −2.6 eV, while a vacancy gives a binding energy of −8.7 eV. Although the vacancy defect significantly possess a greater BE than the cohesive energy of bulk Sc, these set-ups would eventually require a very high concentration of vacancies. It is possible but relatively difficult to achieve a high coverage of Sc on the outer surface of SWCNT in the realms of present technologies.

The increased interatomic distance, compared to C–C bonds for pure ZGNR, further weakens the π-bonding resulting in a buckled structure. This alters the electronic structure by eventually opening the HOMO-LUMO (H-L) gap as n_zig_ increases as displayed in Table 1. As with the case of spin, its H-L gap trend is due to the spatial distribution of edge-state wave functions^22^. The local character of the exchange interaction can also modify the conductivity of NZE-GNR^22^. Ultimately, the conductivity of NZE-GNR decreases due to external distortions accompanied by weak π-bond which regains its stability by puckering. This is further exemplified in Sc edge decorated NZE-3NVGNR, the puckered structure with a dihedral angle in between 171° to 176° as shown in Fig. 2 becomes more stable than the planar 180°. This agrees well with an analogous calculation that demonstrates the energy difference between puckered structures is favored in silicenes^35^. The relationship established here is that the gap size is inversely proportional to n_zig_, which corresponds to a smaller ribbon width. Indeed, the GNRs energy band gap is deeply rooted on the width of GNR. The larger band gaps obtained here with smaller ribbon widths is accounted to quantum confinement effect. The calculations presented here in Table S1 shows that metallic properties also emerges when the nanoribbon width is incrementally increased. The shift from a semiconductor to metal can be attributed, not only to the n_zig_ defects, but also to the size of the ribbon width. Furthermore, the edge formation energy strongly correlates with the HOMO-LUMO gap for nanoribbons. Nanoribbons with small edge formation energy are metals (w ≥ 7) in Table S1, while ribbons with larger formation energy are semiconductors. The Nanoribbons in particular exhibit a sizeable bandgap. Also, Sc functionalization can transform a semiconductor to a metal suggesting potential application of the composite material in electronic devices. The calculated charge transfer (C_Sc_) from Sc atom to the NZE-3NVGNR edge in Table 1 suggests that it carries a positive charge. Although the 3*d* orbitals are filled upon adsorption, it is interesting to note that the overall charge transfer, calculated using the Mulliken analyses, amounts, to nearly 0.205 to 0.316 electron from the Sc atom to the surrounding C atoms. This indicates that the electrons of the Sc atom participate in the Sc-C bond through hybridization with the *p* orbitals of C via a charge transfer mechanism. The partially cationic character of the incorporated Sc decreases as n_zig_ increases. These can facilitate the adsorption of foreign small molecules such as hydrogen gas^20,32^. The second part of the study will show that Sc-dispersed NZE-3NVGNRs have a great potential for hydrogen storage as discussed in the next section.

### Hydrogen Storage of Sc decorated NZE-3NVGNR

The attainment of moderate H_2_ adsorption energy (E_ads_) remains to be a formidable challenge, due to either weak physisorption or strong chemisorption that prevents hydrogen release. As discussed above, a new novel approach was proposed to enhance the storage ability of NZE-3NVGNR based systems by doping it with Sc. According to Kuang He *et al*.^36^ the contraction of C–C bond in graphene edges is attributed to the formation of a triple bond and the absence of H-atom functionalization. The C–C bond compression is in agreement with the triple bond length, indicating that a GNR edge without H-atoms for decoration of TM atoms is achievable. Nevertheless, calculations^32^ show a competition between H and Sc atoms in narrow width nanoribbons, indicating that the edge is eventually populated by H and Sc atoms. However, hydrogen is certainly unstable under electron bombardment; a study shows that Sulphur atoms lend themselves as a more stable side termination^36^. These Sulphur-terminated carbon strips might be the narrowest possible GNRs that are stable. In light of that controlled synthesis, it is expected that fully Sc edge-decorated GNR is possible. A molecular dynamics (MD) calculation performed on the boxed fragment in Fig. 2 suggest that the currently proposed system without H-atom termination is stable. Further, the Sc atoms prefers to adsorb near the edge C atoms. The total energy MD simulations using the velocity form of Verlet algorithm method in eV conducted at 300 K for 3000 steps in Fig. 2d with a time step of 1 fs. For constant-temperature MD the Nose-Hoover thermostat deterministic algorithm controls the temperature of the system. It turns out that the total energy is nearly constant with a −0.1 to +0.1 Å fluctuations in the Sc-C bond length during the entire simulation. Here the results demonstrate that the Sc-based nanomaterials if synthesized are both thermodynamically and mechanically and stable at room temperature. On the other hand, several TMs were considered such as Ti, V and Pt as control groups for comparison to Sc with regards to the (a) best dispersability - ratio between the E_b-Sc_ and E_coh_; (b) biggest charge transferred from TMs to the nanoribbon; an (c) ideal E_ads_ (0.16 ∼ 0.42 eV/H_2_)^30–41^, for reversible adsorption and desorption; (d) largest charge transferred from one H_2_ adsorbed to TM atom, Sc is the TM of choice for achieving efficient H_2_ storage media. The Sc/NZE-3NVGNR model possess high affinity with H_2_ due to the charge transfer from the Sc dopants to the carbon-based GNR with 3NV^17,33–41^ and NZE imperfections within the ribbon can drastically influence the H_2_ binding^11,24^ and eventually gives rise to variation of H_2_ adsorption strength in terms of the average BE as n_zig_ progresses. In order to show how the nano-shape affects the Sc-H_2_ BE, DFT was utilized to predict the largest number of H_2_ attached on Sc/NZE-3NVGNR. This is achieved by intuitively increasing the number of H_2_ molecules from one to five that is attached to the system. As the first H_2_ molecule is adsorbed on Sc metal site located in the Sc/NZE-3NVGNR in Fig. 2, the energy evolution of the system decreases slowly as attractive forces dominate when the hydrogen comes near the vicinity of the Sc. However, as the Sc-H_2_ orbital overlap progresses; the H_2_ molecule is attracted to the Sc atom with an abrupt decrease in energy as it reaches the bottom of the potential energy well.

**Figure 3:**
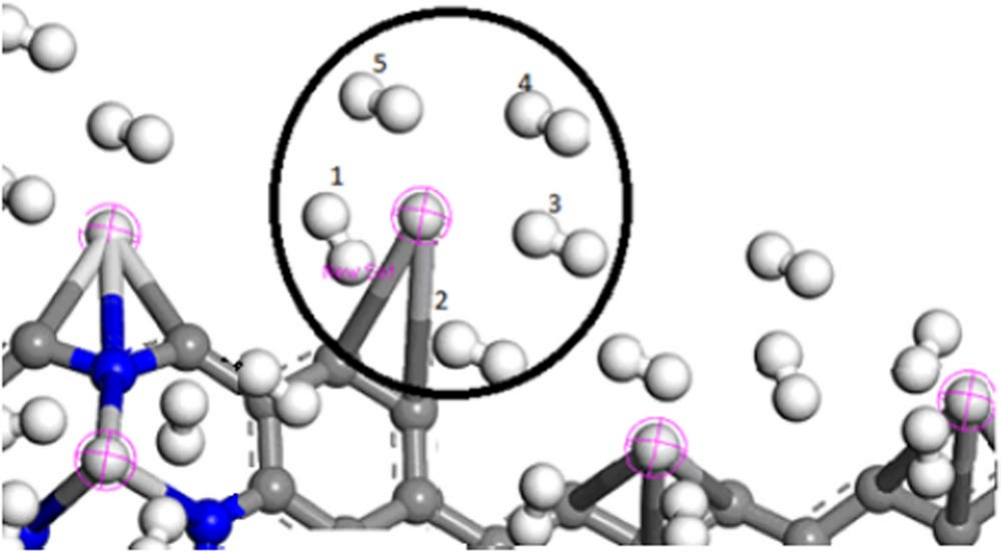
The relaxed structure of Sc/NZE-3NVGNR system with an average of 5H_2_ attached per Sc. The highlighted spheres in pink are Sc atoms, N atom is blue, H_2_ molecule is white and C atom is gray.

The energy gained by the first adsorption starts at −0.249 eV/H_2_ for n_zig_ = 1 to −0.415 eV/H_2_ for n_zig_ = 12 (−0.189 eV/H_2_ for n_zig_ = 1 to −0.337 eV/H_2_ for n_zig_ = 12 with H-atom termination) as shown in Table 1. Interestingly, according to Table S2 the position of the Sc-N_3_ center highlighted in pink in Fig. 1 has a negligible effect on the E_ads_. The E_ads_ is nearly independent with respect to the position of the Sc-N_3_ center. The n^th^ H_2_ E_ads_ in Sc decorated NZE-3NVGNR was calculated by

3 $${{\rm{E}}}_{{\rm{ads}}}={{\rm{E}}}_{{\rm{Sc}}/{\rm{NZE}}-3{\rm{NVGNR}}+{{\rm{nH}}}_{2}}\,-\,{{\rm{E}}}_{{\rm{Sc}}/{\rm{NZE}}-3{\rm{NVGNR}}+({\rm{n}}-1){{\rm{H}}}_{2}}\,-\,{{\rm{E}}}_{{{\rm{H}}}_{2}}$$

where ${{\rm{E}}}_{{\rm{Sc}}/{\rm{NZE}}-3{\rm{NVGNR}}+{{\rm{nH}}}_{2}}$, ${{\rm{E}}}_{{\rm{Sc}}/{\rm{NZE}}-3{\rm{NVGNR}}+({\rm{n}}-1){{\rm{H}}}_{2}}$. ${{\rm{E}}}_{{{\rm{H}}}_{2}}$ are the relaxed total energy of the Sc-decorated NZE-3NVGNR with n H_2_ attached, Sc-decorated NZE-3NVGNR with n−1 H_2_ adsorbed, a single H_2_ adsorbed, respectively. The H-H bond length is elongated upon being physisorbed to the Sc metal and it can go as high as 0.798 Å for n_zig_ = 12 as seen in Table 1 denoted by the average H-H distance (${{\rm{D}}}_{{{\rm{H}}}_{2}}$). The analysis shows that Sc-H_2_ distance of the first added H_2_ molecule is closer to the Sc atom compared to the succeeding H_2_ added to the system. The calculated E_ads_ for n greater than one is summarized in Table 2. The E_ads_ < 0 of the additional hydrogen molecules added are all thermodynamically stable just like in the first adsorption. The succeeding four hydrogen molecules are also physisorbed in the system as suggested in Table 2. In general, the adsorption energy based on GGA/PBE level of theory per H_2_ added to the Sc/NZE- 3NVGNR system increases as n_zig_ progresses (puckered) and decreases as the number of attached H_2_ increases from left to right of the table. Interestingly, this is also the case for a pure ZGNR (flat) decorated with Sc along its edge wherein the E_ads_ also diminishes as the amount of H_2_ attached per Sc increases with a saturated value of five per Sc. A Quantum chemical calculation performed by Datta^41^ on a series of cyclic rings (S_2_N_2_ and S_3_N_3_^*−*^) with and without doping of TM (Ni, Pd and Pt) to study their ability to store hydrogen shows that S_2_N_2_ is planar while S_3_N_3_^*−*^ puckered to C_3v_ symmetry and undoped SN rings and chains are predicted to have dispersive interactions with H_2._ The correlation between n_zig_ and adsorption energy can be established partially as it appears to be deeply rooted with an increased puckered configuration and stability. This correlation is valid if puckering is achieved through small changes in bond lengths and angles compared from the flat ZGNR model^17^. The optimal range of E_ads_ falls between physisorption and chemisorption, which serves as a good tolerance for reversible H_2_ adsorption-desorption. The D_H2_ not tabulated here is also elongated and can be explained by the classical Kubas systems^42,43^. The σ-donation from H_2_ to the Sc metal, π-back-donation from the metal to the H-H dominates, and consequently, this behavior can be traced to a lowering in energy of the metal *3d* orbitals of the Sc atom. To achieve the DOE target, moderate ratio of Sc adsorption sites to the weight of NZE-3NVGNR is highly desired. To satisfy this ratio, the NZE-3NVGNR with n_zig_ = 12 was considered, where both NZE are passivated with Sc atoms as shown in Fig. 3. Here the H_2_ storage capacity of the NZE-3NVGNR is predicted to exceed 9.70 wt% with 5H_2_ attached per Sc on the average^17,33–41^. The Sc-H_2_ distances are on the average about 2.1 Å with a noticeable molecular H_2_ binding with respect to the Sc adsorption sites. The final product is denoted as 60H_2_-Sc_12_/NZE-3NVGNR for n_zig_ = 12 again with both NZE sides populated with Sc-5H_2_ pairs. It can be argued that as a 6^th^ hydrogen molecule is added near the Sc a decent bonding is not possible and it is below the acceptable threshold for physisorbed molecule, which confirms that the system is saturated with 5H_2_ per Sc. The E_ads_ for the 5th H_2_ added (E_ads-5H2_) is −0.283 eV per H_2_ with all hydrogen atoms attached in molecular form with a bond length of around 0.763 Å, which is obtained by averaging all the Sc-5H_2_ bonds. The hydrogen molecules on the GNR are relatively close to each other. Several trials were performed, regardless of the initial positions of the adsorbed H_2_, almost the same final configurations are achieved wherein five H_2_ populate the vicinity of the Sc metal. The corresponding hydrogen molecules are all tilted in a certain degree after optimization, one of the two H atoms prefers to be closer to the Sc site of the 60H_2_-Sc_12_/NZE-3NVGNR assembly.

**Table 2:** Adsorption energy based on the GGA/PBE level of theory per H_2_ added to the Sc/NZE-3NVGNR (E_ads_) incurred by successive increase of n_zig_. Incorporated with vdW and BSSE correction.

n_zig_	E_ads-H2_ (eV)	E_ads-2H2_ (eV)	E_ads-3H2_ (eV)	E_ads-4H2_ (eV)	E_ads-5H2_ (eV)
1	−0.249	−0.183	−0.190	−0.201	−0.159
2	−0.256	−0.226	−0.236	−0.201	−0.165
3	−0.257	−0.245	−0.257	−0.231	−0.175
4	−0.307	−0.242	−0.259	−0.220	−0.161
5	−0.251	−0.246	−0.251	−0.201	−0.177
6	−0.283	−0.237	−0.233	−0.205	−0.194
7	−0.254	−0.234	−0.220	−0.164	−0.165
8	−0.275	−0.215	−0.213	−0.175	−0.163
9	−0.283	−0.257	−0.224	−0.164	−0.171
10	−0.312	−0.297	−0.254	−0.199	−0.191
11	−0.356	−0.363	−0.312	−0.265	−0.227
12	−0.415	−0.413	−0.404	−0.370	−0.283

In reality, E_ads_ should increase during adsorption and decrease during desorption for an ideal hydrogen storage system. These two seemingly contradictory requirements are a problem that can be addressed upon application of an external electric field (EF). Electric fields were applied perpendicular to the nanoribbon plane (upward defined as “+” and downward defined as “−”). An EF of +0.20 V/Å applied to the NZE reduces the charge on Sc and the adsorption energy of H_2_ decreases. On the other hand, upon applying an EF of −0.20 V/Å to the NZE, the charge on Sc increases, and the adsorption energy of H_2_ is enhanced. The application of a (+) electric field yields a decrease in the E_ads_ by 0.085 eV during desorption while a (−) electric field increases the E_ads_ by 0.086 eV during adsorption. The adsorption energy per H_2_ of the 60H_2_-Sc_12_/NZE-3NVGNR is also sensitive to the direction and magnitude of the applied EF. The applied electric field is proven here as an effective means to redistribute the charges in the Sc atoms. In particular, the GNR was shown to exhibit a pronounced response to an external electric field. Since the magnitude and direction of the electric field can readily be changed, the bonding in the adsorption and desorption process can be dictated. The electric field can act as a switch^44–48^ for H_2_ storage media. In light of DFT calculations of narrow NZE-3NVGNR using LDA-PWC, the average E_ads_ of H_2_ is also in good agreement within the adsorption-desorption range of hydrogen storage at 298 K (0.20 ∼ 0.70 eV/H_2_)^43,45,49^.

Also, the high Sc atom density further increases the adsorption energy of H_2_ as presented in Table S3 is essential in achieving the DOE efficiency. The proposed model provides the maximum wt. % H_2_ density that can be adsorb in the nanocomposite material theoretically. Potentially it can surpass the benchmark required for system-weight efficiency set by the DOE in designing composite materials for H_2_ storage. This is possible by decreasing the width of the GNR coupled to saturating the edges with the lightest transition metal. All of the above discussion clearly demonstrate that current Sc ratio to the NZE-3NV imperfections of the GNR is an excellent choice for hydrogen storage purposes. Lebon *et al*.^50^ performed DFT calculations to investigate the adsorption of H_2_ on Ti-doped ZGNRs using a nonlocal vdW functional. The Ti atom is adsorbed at a central or lateral hole site, each Ti atom can bind up to four H_2_ molecules. On this basis the effective H_2_ coverage on Ti-coated GNRs is beyond the DOE target of 6 wt. %. The magnitude of the H_2_ adsorbed is 0.22–0.40 eV/H_2_. Tranca *et al*.^51^ also calculated the adsorption of metal atoms (Mg, Ca, Ti) on armchair GNRs with O/-OH saturated edges and its interaction. The magnitude of the H_2_ adsorbed is between 0.15–0.98 eV/H_2_. Usual hydrogen storage materials need a modulated adsorption energy values (0.16–0.42 eV/H_2_)^52,53^ which guarantees both adsorption and desorption processes such as the proposed Sc/NZE-3NVGNR system, suitable for practical H_2_ storage applications. The intricate roles of the NZE imperfections and pyridine defects in Sc functionalized NZE-3NVGNR in its energetics and H_2_ adsorption is finally address. Furthermore, the electronic and magnetic properties of these nanoribbons are fine-tune by controlling the shape of the ribbon edges, demonstrating that the NZE-3NVGNR is an interesting material for spintronic devices.

**Figure 4:**
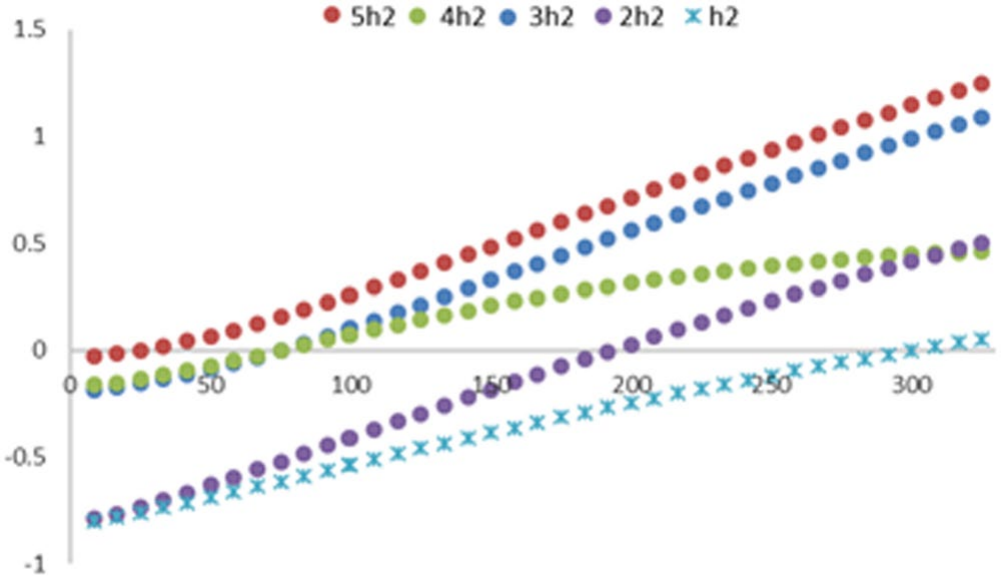
The ∆G for the process C_38_N_3_Sc_10_ + n(10H_2_) → C_38_N_3_Sc_10_(10H_2_)_n_ for n = 1 to 5. The temperatures at which ∆G = 0 eV/H_2_ starting at 1 to 350 K are critical temperatures.

Finally, we end this paper by providing an estimate what conditions are required for the system to adsorb or desorb H_2_ molecules. The effect of temperature on ∆G for a smaller cluster enclosed in the rectangular area presented Fig. 2 serves as a model. For the reaction process C_38_N_3_Sc_10_ + n(10H_2_) → C_38_N_3_Sc_10_(10H_2_)_n_, calculating the contributions of ground state energy and entropy of the reactants and product provides the opportunity to fully determine the Gibbs free energy at any given temperature Т. The total entropy (**S**) is expressed as the sum of the translational (S_t_), rotational (S_r_), vibrational (S_v_) and electronic (S_e_) entropy components. The **S** is incorporated to the formula **H**[C_38_N_3_Sc_10_(10H_2_)_n_] − T·**S**[C_38_N_3_Sc_10_(10H_2_)_n_] to find the free energy of the product (G_p_). The familiar enthalpy (**H**) term is constructed in an analogous way as **S** derived from statistical mechanics. The same formulation when applied to C_38_N_3_Sc_10_ and H_2_ yields the free energy of the reactants (G_r_). In general, ΔG = G_p_ − G_r_ so the ∆G = {G[C_38_N_3_Sc_10_(10H_2_)_n_] − nG[10H_2_] − G[C_38_N_3_Sc_10_]}/n for n = 1 to 5 to estimate the maximum reversible hydrogen storage capacity at 1 atm. 50 H_2_ molecules can be spontaneously adsorbed on C_38_N_3_Sc_10_ below 40 K As shown in Fig. 4. Along with the rise of temperature, it releases two additional H_2_ at temperatures around 77 K. When the temperature increases up to 298 K, the last H_2_ molecules are desorbed. All of the four H_2_ are released at room temperature, 40H_2_ in the C_38_N_3_Sc_10_(10H_2_)_5_ complex can be readily adsorbed at 77 K and desorbed incrementally as it approaches 298.15 K under atmospheric pressure. The calculation is accurate because proper electron correlation effects is accounted, as the molecular hydrogen interaction is weak in nature. We have used Grimme’s method^54^ for obtaining the dispersion correction for all hydrogen interaction energies. The method developed by Grimme is widely available and can be combined with various commonly used DFT functionals. A systematic study of techniques^55^ for evaluating noncovalent interactions within DFT is presented through comparison of binding energy for modeling adsorptive Hydrogen storage. In particular, the capacity for producing a desired result of functionals to encompass long-range forces. The importance of DFT + dispersion corrections was assessed against reference interaction energies. Specifically Grimme’s method provided sufficiently accurate results^56^, which indicates that, the maximal reversible hydrogen storage densities greater than 7.70 wt. %, well above the DOE target is achievable.

## Conclusion

The electronic and magnetic properties of NZE-3NVGNR was studied using DFT. The calculation shows that the existence of short-range magnetic order and magnetism is closely linked to the structure of the zigzag edges of the ribbon. The results indicate that even in the presence of a high defect density, for overall zigzag oriented edges the spin polarization decreases as the corresponding width of the ribbon narrows. The 3NV and the NZE imperfections in GNR caused an enhanced chemical functionalization of Sc with reduced clustering over the metal decorated NZE-3NVGNR. Strong binding of five hydrogen molecules per Sc site to the composite material Sc/NZE-3NVGNR was observed that is highly tunable through the application of an external electric field. The binding and release of the hydrogen molecule to the Sc/NZE-3NVGNR assembly turns out to be favorable at the LDA and GGA level. The predicted hydrogen storage densities greater than 7.70 wt. % at 298.15 K under atmospheric pressure is realizable.

## Electronic supplementary material

Supplementary Information
