# Supplementary material for: Hydrogen Adsorption on Nearly Zigzag-Edged Nanoribbons: A Density Functional Theory Study
Source: Sci Rep. 2017 Nov 16;7:15727. doi: 10.1038/s41598-017-14189-z (PMC5691176; doi:10.1038/s41598-017-14189-z)
Supplement: Supplementary file 1 — Supplementary Information [file 41598_2017_14189_MOESM1_ESM.doc]

**Supplementary Information**

**Hydrogen Adsorption on Nearly Zigzag-Edged Nanoribbons: A Density Functional Theory Study**

**Michael Rivera Mananghaya, ^1, 3^** * **Gil Nonato Santos^2^ and Dennis Yu^2^**

**^1^**Ateneo de Manila University, Katipunan Ave, Quezon City, 1108 Metro Manila, Philippines.

**^2^**De La Salle University, 2401 Taft Avenue, 0922 Manila, Philippines.

**^3^**NRCP (IX), DOST, Gen. Santos Ave., Bicutan, Taguig City 1631, Philippines.

*Corresponding author e-mail: *mikemananghaya@gmail.com*

**Supplementary Table S1**. The edge formation energy (E_edge_), HOMO-LUMO gap (E_gap_) and adsorption energy (E_ads_) incurred by successive increase of *w*, where *w* denotes the label for a GNR with *w* zigzag chains as *w*-ZGNR.

| *w*-ZGNR | E_edge_  (meV/edge) | E_gap_  (eV) | E_ads_  (eV/H_2_) |
| --- | --- | --- | --- |
| 3-ZGNR | 150.654 | 0.130 | -0.277 |
| 4-ZGNR | 100.123 | 0.081 | -0.276 |
| 5-ZGNR | 95.861 | 0.020 | -0.281 |
| 6-ZGNR | 91.841 | 0.010 | -0.283 |
| 7-ZGNR | 87.114 | - | -0.284 |
| 8-ZGNR | 85.837 | - | -0.286 |
| 9-ZGNR | 83.494 | - | -0.288 |
| 10-ZGNR | 82.406 | - | -0.290 |

**Supplementary Table S2**. The edge formation energy (E_edge_) and adsorption energy (E_ads_) incurred with respect to the position of the Sc-N_3_ center.

| Sc-N_3_ center location | E_edge_  (meV/edge) | E_ads_  (eV/H_2_) |
| --- | --- | --- |
| 1 | 101.019 | -0.249 |
| 2 | 103.613 | -0.249 |
| 3 | 106.467 | -0.250 |
| 4 | 109.321 | -0.248 |
| 5 | 112.170 | -0.250 |
| 6 | 120.201 | -0.252 |
|  |  |  |

**Supplementary Table S3**. The Adsorption energy (E_ads_) per H_2_ of the Sc/NZE-3NVGNR complex incurred by successive increase of n_zig_ at 33 and 18 wt. % Sc.

| n_zig_ | E_ads-5H2_ (18 wt. %)  (eV) | E_ads-5H2_ (33 wt. %)  (eV) |
| --- | --- | --- |
| 1 | -0.153 | -0.159 |
| 2 | -0.158 | -0.165 |
| 3 | -0.168 | -0.175 |
| 4 | -0.154 | -0.161 |
| 5 | -0.176 | -0.177 |
| 6 | -0.185 | -0.194 |
| 7 | -0.158 | -0.165 |
| 8 | -0.156 | -0.163 |
| 9 | -0.164 | -0.171 |
| 10 | -0.183 | -0.191 |
| 11 | -0.213 | -0.227 |
| 12 | -0.272 | -0.283 |


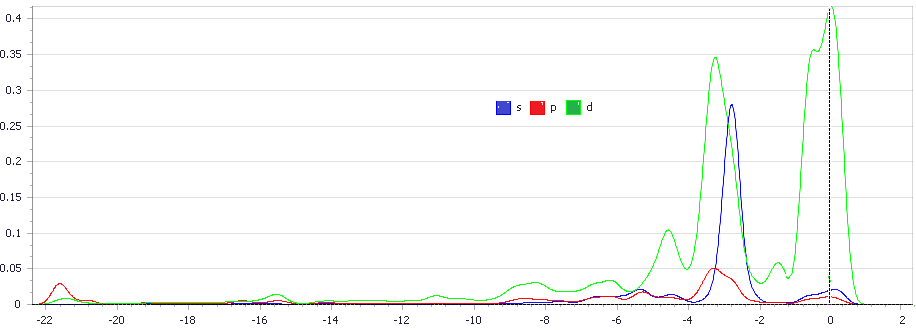


**Supplementary Figure 1.** The Partial Density of States of the Sc directly bonded to C of the NZE-3NVGNR. The unit of the vertical axis is in electrons/eV and the horizontal axis is in eV.
